# Pregnancy rhinitis in Turkish women: Do gestational week, BMI and parity affect nasal congestion?

**DOI:** 10.12669/pjms.324.10164

**Published:** 2016

**Authors:** Burak Ulkumen, Burcu Artunc Ulkumen, Halil Gursoy Pala, Onur Celik, Nevin Sahin, Gizem Karaca, Meltem Demirdag

**Affiliations:** 1 Burak Ulkumen, Assistant Professor, Department of Otorhinolaryngology, Celal Bayar University, School of Medicine, Manisa, Turkey; 2 Burcu Artunc Ulkumen, Associate Professor, Department of Obstetrics and Gynecology, Celal Bayar University, School of Medicine, Manisa, Turkey; 3 Dr. Halil Gursoy Pala, Department of Obstetrics and Gynecology, Celal Bayar University, School of Medicine, Manisa, Turkey; 4 Prof. Onur Celik, Professor, Department of Otorhinolaryngology, Celal Bayar University, School of Medicine, Manisa, Turkey; 5 Dr. Nevin Sahin, Department of Otorhinolaryngology, Celal Bayar University, School of Medicine, Manisa, Turkey; 6 Dr. Gizem Karaca, Department of Otorhinolaryngology, Celal Bayar University, School of Medicine, Manisa, Turkey; 7 Dr. Meltem Demirdag, Department of Otorhinolaryngology, Celal Bayar University, School of Medicine, Manisa, Turkey

**Keywords:** Pregnancy rhinitis, Nasal congestion, Rhinitis, Incidence

## Abstract

**Objective:**

To determine the cumulative incidence of pregnancy rhinitis along with prevalence in different trimesters and to find out whether gestational age, BMI and parity have any effect on pregnancy related nasal congestion.

**Methods:**

In the prospective protocol at the obstetrics outpatient clinic, 167 pregnant women were enrolled consecutively. According to exclusion criteria, 67 of them were excluded. Visual-Analogue-Scale (VAS), Nasal-Obstructive-Symptom-Evaluation (NOSE) scale and Discharge-Inflammation-Polyps/Oedema (DIP) scoring were utilized for diagnosis of pregnancy rhinitis. Besides, weight, length, age, parity and week of pregnancy were recorded.

**Results:**

Total prevalence of pregnancy rhinitis was 17.17% and cumulative incidence was 38.89%. Our study revealed significant relation of NOSE score with both gestational week (r=0.474, p=0.001) and BMI (r=0.301, p=0.003). VAS score was significantly related with gestational week (r=0.409, p=0.001) and BMI (r=0.270, p=0.007). DIP score was found to be correlated only with gestational week (r=0.375, p=0.001).

**Conclusion:**

Cumulative incidence of pregnancy rhinitis was 38.89%. Nasal congestion was significantly associated with BMI and gestational week. Patients should be informed about unfavorable fetal and maternal outcomes of pregnancy related nasal congestion which is triggered by obesity and excessive weight gain in pregnancy.

## INTRODUCTION

Pregnancy rhinitis (PR) is a quite common condition whose pathophysiology has not been fully described. In contrast to its relatively high incidence PR has a relatively low awareness level among public when compared with other nasal pathologies. It may lead to snoring and sleep apnea syndrome which may trigger some serious maternal (hypertension, preeclampsia) and fetal (low Apgar score, intrauterine growth retardation) complications.^1^-^4^

PR is defined as nasal congestion with rhinorrhea and sneezing which arise typically during pregnancy and resolve within three weeks after birth without any known history of allergy and other nasal pathologies (septal deviation, polyposis, sinusitis, etc.).^5^ There is no consensus on its etiology. Some researchers suggest that the PR occur due to aggravation of subclinical allergy^5^,^6^ while majority claim the rising serum levels of hormones like progesterone, estrogen and placental growth hormone.^3^

Incidence of PR has been reported as between 9% - 40% which is a quite wide range.^1^,^7^,^8^ Although it can begin in any trimester it’s prevalence has been reported far much higher in the 3th trimester.^9^ This feature necessitates prospective cohort studies for detection of cumulative incidence.

We aimed to determine the cumulative incidence of PR along with prevalence in different trimesters in Turkish women. We also tried to find out whether maternal age, gestational week, BMI and parity have any effect on pregnancy related nasal congestion.

## METHODS

A prospective observational cohort study was performed. This research received approval from the institutional review board, Celal Bayar University Medical Ethics Committee. All patients signed informed consent and they were informed about the study. One hundred sixty seven pregnant women were enrolled consecutively in the prospective protocol at the obstetrics outpatient clinic according to strict inclusion and exclusion criteria. The inclusion criterion was pregnant women who come to the obstetrics outpatient clinic for routine follow-up. The exclusion criteria were any history of allergy, upper airway infection, nasal obstruction before pregnancy and purulent nasal discharge in endoscopic nasal examination.

**Fig.1:**
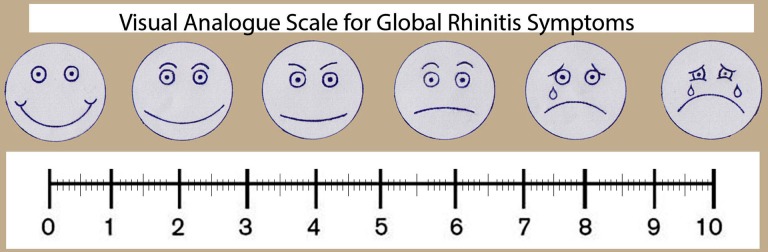
Visual Analogue Scale (VAS).^7^

The enrollment period was June 2, 2014 through July 26, 2015. All participants were asked to complete Visual Analogue Scale (VAS) (Fig.1) and Nasal-Obstructive-Symptom-Evaluation (NOSE) scale (Table-I) whose sensitivity and specificity were validated by previous studies.^10^,^11^ They complete the NOSE scale as indicated by circling the response closest to describing their current symptoms. Answers were summed and multiplied by five to base the scale out of a possible score of a 100 for analysis. We assumed the cut off value as 5 cm for VAS and 45 points for NOSE score in the light of previous studies.^12^,^13^

**Table-I:** The NOSE (Nasal Obstruction Symptom Evaluation) scale.^8^

Over the past 1 month how much of a problem were the following conditions for you? Please circle the most correct response					

	Not a problem	Very mild problem	Moderate problem	Fairly bad problem	Severe problem
1. Nose obstruction and stuffiness	0	1	2	3	4
2. Nose obstruction	0	1	2	3	4
3. Trouble breathing through my nose	0	1	2	3	4
4. Trouble sleeping	0	1	2	3	4
5. Unable to get enough air through my nose during exercise or exertion	0	1	2	3	4

Then all of the participants were examined by an otorhinolaryngologist who was blinded to the NOSE and VAS scores. To quantify the findings of nasal examination we ask the physician to complete a relatively novel scale (primarily designed for nasal polyposis) called Discharge-Inflammation-Polyps/Oedema (DIP) scale (Table-II) which is also validated by previous studies.^9^,^10^ As might be expected there was not any participant with nasal polyposis (owing to exclusion criteria) in our study population. Therefore, we grade the 3^rd^ parameter (Polyps/Edema) only by evaluating the oedema.

**Table-II:** The DIP (Discharge Inflammation Polyps/Edema) scoring system.^9^

	Absent	Moderate	Severe
1. Discharge	0	5	10
2. Inflammation	0	5	10
3. Polyps/Edema	0	5	10

Weight, length, age, parity and week of pregnancy were recorded. After completion of patient (VAS, NOSE) and physician (DIP) centered quantitative scales we excluded 67 patients. This left us with 100 patients. 31 of them were in the 1^st^ trimester while 32 and 37 of them were in the 2nd and 3^rd^ trimester, respectively.

Patients with VAS ≥5cm and NOSE scale ≥45 points were assumed as candidates for PR. We asked all the patients to come at postpartum 3th week. Of the 100 patients 18 did not come to follow up. The remaining 82 patients were again asked to complete VAS and NOSE scale. DIP score was also completed by the same otorhinolaryngologist according to the endoscopic findings. We determine the prevalence of PR for every trimester by considering the postpartum NOSE, VAS and DIP scores. If the scores of PR candidates at postpartum 3th week were still above the cut off levels for each scale they were approved as having PR. Additionally, we study if maternal age, gestational week, BMI and parity have any impact on PR.

The data are presented as mean ± SD. The spearmen test was used for evaluation of the effect of maternal age, gestational week, BMI and parity on pregnancy related nasal congestion whose intensity was determined by NOSE, VAS and DIP. Significant differences were established at a level of p=0.05 (IBM SPSS Statistics for Windows, Version 21.0. (Armonk, NY: IBM Corp))

## RESULTS

Of the 100 patients; 31 were in the 1^st^ trimester, 32 were in the 2nd trimester and 37 were in the 3^rd^ trimester. The mean age of pregnant women in the study was 29.08±5.59 (range: 17-44); the mean BMI was 25.34±2.76 (range: 18.49-31.35); the mean parity was 1.38±1.00 (range: 0-4); the mean gestational week was 22.79±10.72 (range: 5-41); the mean BMI was 25.34±2.77 (range: 18.49-31.35). 24 patients had a NOSE score greater than 45 or VAS score greater than five at first evaluation. Of these 24 patients; 21 had both NOSE and VAS scores equal or bigger than cut-off levels. one patient had a NOSE score of 45 while her VAS score was three. The remaining two patients both had VAS scores of 5 while their NOSE scores were 30 and 20 respectively. 21 patients -whose VAS and NOSE scores were above cut off levels- were assumed as candidates for PR. one of them –whose first evaluation was done in the 3^rd^ trimester- did not come to postpartum follow up. Seventeen of the 21 PR candidates who had both NOSE and VAS scores below the cut-off levels at postpartum 3^th^ week were diagnosed as PR. Concerning these 17 patients; three of them had first evaluated in the 2^nd^ and 14 of them had first evaluated in the 3^rd^ trimester while no one of these candidates had seen in the 1^st^ trimester.

Accordingly the prevalence of PR for each trimester was specified: 0% in the 1^st^ trimester, 9.38% in the 2^nd^ trimester and 38.89% in the 3^rd^ trimester. Total incidence has determined according to the third trimester group because PR commonly become clinically visible only during the third trimester with having no symptoms during the first and second trimester. Thus we ignore the 1^st^ and 2^nd^ trimester while calculating the total incidence. Total prevalence was 17.17% and the cumulative incidence was 38.89%.

Correlation analysis revealed a significant relation of NOSE score with both gestational week (r=0.474, p=0.001) and BMI (r=0.301, p=0.003), but no significant relation with parity (r=0.145, p=0.155) and maternal age (r=0.051, p=0.620). Similarly, VAS score was significantly related with gestational week (r=0.409, p=0.001) and BMI (r=0.270, p=0.007), but has no significant relation with parity (r=0.077, p=0.452) and maternal age (r=0.046, p=0.655). DIP scale was significantly related with gestational week (r=0.375, p=0.001). In contrast to VAS and NOSE scales, DIP score was significantly related with parity (r=0.231, p=0.021). There was no relation between DIP scale and “BMI (r=0.174, p=0.087) and maternal age (r=0.094, p=0.359)”. The distribution of NOSE, VAS and DIP scores according to the gestational week is seen at the Table-I.

## DISCUSSION

Pregnancy rhinitis (PR) becomes clinically visible almost always during the third trimester with having no explicit symptoms during the first and second trimester. Nevertheless, symptoms may also begin in the 1^st^ and 2^nd^ trimester in a limited number of patients.^9^,^14^ This characteristic of PR necessitates prospective cohort studies for detection of cumulative incidence. The incidence of PR was reported with a wide range of 9% to 40% in different studies.^5^,^7^,^8^ The most comprehensive study was done by Ellegard et al. including 599 Swedish pregnant women in which they found the incidence as 22% by using a questionnaire during the routine pregnancy follow-up.^15^ We, on the other hand found the cumulative incidence as 32.43% which is quite higher. We also found that the PR was most commonly seen during the 3.trimester in line with the previous studies. Besides, a considerable amount of patients also emerged during the 2^nd^ trimester while there was no PR case in the 1^st^ trimester. The prevalence was 0% in the 1^st^, 9.38% in the 2^nd^ and 38.89% in the 3^rd^ trimester, respectively. In the view of these findings, we can say that some asymptomatic patients in the 1^st^ and 2^nd^ trimester may develop PR in the 3^rd^ trimester thus accurate detection of cumulative incidence can only be done by evaluating the pregnancies at least during the third trimester and three weeks after delivery. In fact it is ideal to follow up all pregnant women from the beginning of pregnancy up to postpartum 3^rd^ week. Misinterpretation of the results would be likely in cross-sectional designed studies. In our study, we evaluate the pregnant women twice; first during their routine pregnancy follow up and second at postpartum 3^rd^ week.

**Fig.2:**
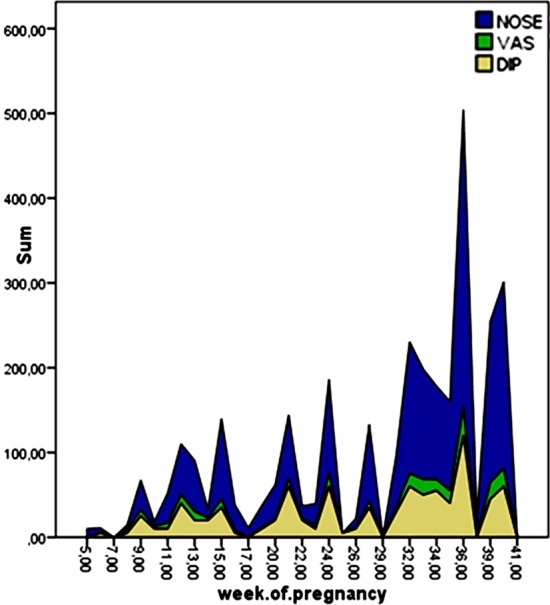
Distribution of NOSE, VAS and DIP scores in relation to the gestational week.

Estrogen, progesterone, human chorionic gonadotropin hormone (HCG), human placental lactogen (HPL) and placental growth hormone (PGH) whose levels are known to be gradually rise throughout the pregnancy have been evaluated in different studies for a possible association with PR. In some of these studies the authors suggested a connection between the aforementioned hormones an PR by studying the relationship between nasal congestion and menstrual cycle (or oral contraceptive intake).^16^,^17^ To the best of our knowledge the effect of estrogen, HCG and HPL on PR has not been studied directly. Ellegard et al found a significant relation between PGH levels and PR. They also showed that there was no relation between progesterone levels and PR.^18^ Our study revealed that nasal congestion was significantly related to gestational week (Fig.2). From this point of view, we suggest that this relevance can be attributed to the pregnancy hormones which are known to be increasing gradually throughout the pregnancy.

No specific test or laboratory tool is available for the diagnosis of PR. It can only be diagnosed depending on history, symptoms, physical examination and exclusion of other possible nasal pathologies (allergic rhinitis, infection, vasomotor rhinitis, septal deviation, polyposis, etc.). In other words it’s a diagnosis of exclusion depending majorly on subjective findings. Nasal obstruction can be evaluated by subjective (VAS, NOSE) or objective (acustic rhinometry, rhinomanometry) means while findings of nasal examination can only be quantified by subjective scales like DIP scoring. VAS and NOSE questionnaires (whose sensitivity and specificity were validated in different nasal pathologies) are patient-centered tests which can quantify the grade of nasal congestion quite successfully.^10^,^11^ DIP, on the other hand is a relatively new physician-centered test which was defined primarily for the evaluation of nasal polyposis as a modification of Lund-Kennedy scoring.^19^,^20^ We prefer to use VAS and NOSE scores for the evaluation of nasal obstruction and DIP score for the quantification of nasal endoscopic findings.

We revealed a significant correlation between VAS, NOSE and DIP scores with gestational week (Fig.2). However, in view of these results, we can propose that increasing levels of estrogen, HCG, HPL, PGH with the advancing gestational week can play a major role in the pathophysiology of PR. But, to work up a direct connection between these hormones and PR, studies measuring the blood levels of estrogen, HCG, HPL and PGH throughout the gestation should be done. We also found a strong relation between pregnancy related nasal congestion (NOSE, VAS) and BMI. We also studied if parity, BMI and age have any possible effect on nasal congestion during pregnancy. To the best of our knowledge our study is the first one analyzing the effect of parity, BMI and age on PR. We found that increased BMI has a major impact on nasal congestion during pregnancy. From this point of view, we can also state that multiple pregnancies (due to relatively increased pregnancy hormones), women with gestational diabetes and obese pregnancies would have increased risk of developing PR.

Nasal congestion may have also a potential risk factor for the proper development and growth of the fetus by causing gradual decrease in oxygenation. Therefore, the treatment of PR is also important for wellbeing of the fetus. Elevation of head at about 30-45 degree during sleep, nasal lavage, oral or intranasal steroid usage are the treatment modalities.^1^

## CONCLUSION

We found the cumulative incidence of PR as high as 32.43%. Nasal congestion was significantly associated with BMI and gestational week. Maternal age and parity had no effect on the nasal congestion. Due to possible restrictive effect on the fetal optimal growth; proper preventive measures are to be undertaken. Patients should be aware of maternal and fetal negative effects of obesity and excessive weight gain in pregnancy.
